# Misalignment of plastic and evolutionary responses of lifespan to novel carbohydrate diets

**DOI:** 10.1098/rsos.231732

**Published:** 2024-01-17

**Authors:** Vikram P. Narayan, Nidarshani Wasana, Alastair J. Wilson, Stephen F. Chenoweth

**Affiliations:** ^1^ School of the Environment, The University of Queensland, St. Lucia, Queensland 4072, Australia; ^2^ College of Life and Environmental Sciences, University of Exeter, Penryn, Cornwall TR10 9FE, UK

**Keywords:** lifespan, evolution, plasticity, adaptation, carbohydrate, diet

## Abstract

Diet elicits varied effects on longevity across a wide range of animal species where dietary discordance between an organisms' evolutionary and developmental dietary history is increasingly recognized to play a critical role in shaping lifespan. However, whether such changes, predominantly assessed in a single generation, lead to evolutionary shifts in lifespan remains unclear. In this study, we used an experimental evolution approach to test whether changes in an organisms' evolutionary and developmental dietary history, specifically carbohydrate content, causes lifespan evolution in *Drosophila serrata*. After 30 generations, we investigated the evolutionary potential of lifespan in response to four novel diets that varied systematically in their ratio of carbohydrate–protein content. We also examined developmental plasticity effects using a set of control populations that were raised on the four novel environments allowing us to assess the extent to which plastic responses of lifespan mirrored adaptive responses observed following experimental evolution. Both high- and low-carbohydrate diets elicited plastic effects on lifespan; however, the plastic responses for lifespan to developmental diets bore little resemblance to the evolved responses on evolutionary diets. Understanding the dietary conditions regulating the match/mismatch of plastic and evolved responses will be important in determining whether a particular match/mismatch combination is adaptive for lifespan. While the differences in evolutionary diet by developmental diet interactions are only beginning to be elucidated, this study lays the foundation for future investigations of carbohydrate contributions to evolved and plastic effects on health and lifespan.

## 1. Introduction

Diet is a fundamental component of an organism's environment, responsible for physiological and morphological adaptations [1]. Variation in dietary macronutrient balance, specifically the ratio of protein to carbohydrate, has been shown to have strong influences on the important life-history trait of lifespan. For example, in insects, diets low in protein and high in carbohydrate typically extend lifespan [2–6]. Further, lifespan is maximized at different macronutrient ratios in males and females indicating fundamental differences in resource acquistion and allocation between the sexes [7–15]. It is worth noting that while sex differences in reproductive performance and fitness are reported by numerous studies, only a very limited number of studies actually demonstrated that males and females differed in their nutritional optima for lifespan [16,17]. To date, most of what is known about the effects of diet on longevity, especially its extension, comes from studies manipulating nutrient content within a single generation [18]. A smaller number of studies have shown that diet-induced lifespan changes can persist through transgenerational plasticity for a small number of generations [19–24]. Experimental evolution has also revealed that lifespan evolves during adaptation to novel diets [20,22]. However, it is not yet known whether lifespan changes seen during experimental evolution mirror those observed in studies of phenotypic plasticity.

Broadly, it is well recognized that genotypic evolution and phenotypic plasticity are two key mechanisms by which natural populations respond to environmental change; however, their relative contributions remain unresolved [25–28]. For instance, phenotypic plasticity is generally assumed to be adaptive, allowing the phenotype to move in the direction of higher fitness. However, plasticity can also be maladaptive, moving the phenotype in the opposite direction to adaptive evolution when organisms are exposed to unusual or novel environments [29,30]. In the context of diet and lifespan, recent studies in *Drosophila* have demonstrated the evolutionary potential for lifespan to evolve genetically under different dietary conditions; however, the extent to which evolutionary and plastic responses align in response to diet remains unclear [20,22]. In this study, we combine experimental evolution and phenotypic plasticity assays to bridge this gap in our understanding. Long-term adult health and lifespan is shaped by both current diet and past dietary history, where persistent long-term effects of nutrition transmitted from parent to offspring are known to programme lifespan [31]. These effects, including caloric or dietary restriction (DR), can range from being instantaneous to inter-generational in nature [32]. In *Drosophila*, flies switched from DR to an ad libitum diet later in life immediately adopted the survival pattern of the group kept on the ad libitum diet treatment [33]. In murine models, switches between DR and unrestricted feeding have shown mixed results, but the results of a meta-analysis do suggest the presence of a lasting ‘memory' of diet [34].

**Figure 1.**
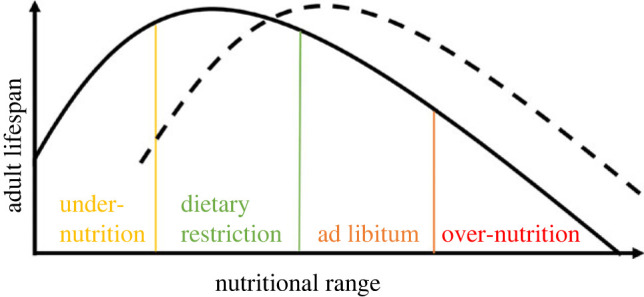
Lifespan reaction norm to diet. Diet nutritional content has a convex shaped relationship with lifespan, ranging from under-nutrition, dietary restriction and maximal lifespan to ad libitum feeding and over-nutrition. The lifespan reaction norm can shift or change shape (dashed line) in response to genetic or environmental changes. For instance, dietary restriction effects can lead to increased lifespan on the focal curve but under-nutrition on the dashed curve. Adapted from [35].

Disproportionate dietary sucrose has been used to create a fly model of an obesogenic and unhealthy diet that bears relevance to modern human diets; however, different hypotheses for the evolution of lifespan under a high-carbohydrate diet have rarely been tested, and a capacity for lifespan to evolve in response to macronutrients is yet to be established for many model systems (but see [20,22]). Diet concentrations have been shown to have a bell-shaped relationship with lifespan, ranging from under-nutrition to over-nutrition (figure 1).

**Figure 2.**
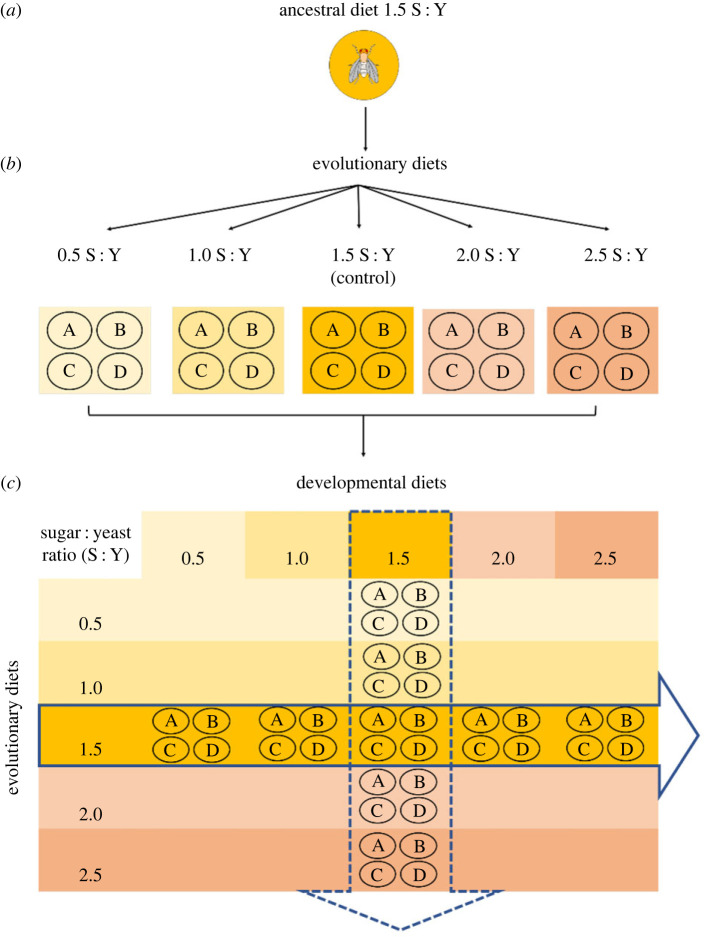
Experimental evolution design where (*a*) *D. serrata* flies from an outbred and long-term laboratory-adapted population were evolved on five distinct diets with different sucrose content as adults for 30 generations (electronic supplementary material, table S1). (*b*) Flies from each diet in the experimental evolution experiment were kept as four independent replicates. (*c*) To remove any parental effects from the evolution diet treatments, experimental flies were subjected to two generations of common garden before we used 10 replicate vials with 10 flies per replicate for each evolution diet (*n* = 400) to test for evolved and plastic lifespan responses. The solid blue arrow represents the experimental set-up for examining the plastic responses to developmental diet, i.e. the transfer of the ancestral diet replicate populations lines to every other developmental diet. The dotted blue arrow represents the experimental set-up for examining the evolved changes, i.e. the transfer of replicates from every evolutionary diet back to the ancestral diet.

Much of our current understanding of how diet alters lifespan comes from studies using model organisms where diet has been manipulated by changing the proportions of protein relative to carbohydrate. Diets with lower protein content and higher carbohydrate content have been associated with increased lifespan and healthspan in *Drosophila*. However, little is known about the effects of carbohydrate–protein diets on longevity phenotypes in response to manipulating the carbohydrate concentration against a fixed nutritional background. To determine if the response of lifespan to changing carbohydrate : protein ratios follow the same pattern as changing protein : carbohydrate ratios, we created four novel diets where we varied the sucrose-to-yeast (S : Y) ratio by manipulating carbohydrates (sucrose) while keeping protein (yeast) content fixed (figure 2). Our approach to diet manipulation was important for two reasons. First, carbohydrate-rich diets represent a fitting model of unhealthy, modern, human diets, and the carbohydrate diets used in this experiment are a particularly relevant example of recent nutritional changes in many human populations with important implications for human health [36–38]. Second, the effects of a high carbohydrate (sucrose) manipulation in diet have not yet been studied extensively, though consistent with our findings, studies have shown that adults developed on high-carbohydrate diets have reduced lifespan [39,40].

In this study, we used an outbred population of *Drosophila serrata* [41] that had adapted for over 200 generations to laboratory culture in a diet where the carbohydrate content is 1.5 times that of the protein content. For 30 generations, we exposed replicates of the founding population to these four novel diets spanning a carbohydrate–protein gradient to detect evolved responses in lifespan. To elucidate developmental plasticity effects, we concurrently assayed lifespan in a set of control populations that were raised on the four novel environments. This comparative approach allows us to assess the extent to which plastic responses of lifespan mirrored adaptive responses observed following experimental evolution. While we found both high- and low-carbohydrate diets to elicit plastic and evolved effects on lifespan, the plastic responses to developmental diets bore little resemblance to the evolved responses on evolutionary diets indicating that plastic responses to reduced sucrose diets may initially be maladaptive for lifespan.

## 2. Methods

### 2.1. Fly husbandry

Flies used to set up the experimental evolution population experiment were all derived from a wild-type, outbred and long-term laboratory-adapted population that was founded from 102 inbred lines of the *D. serrata* Genomic Reference Panel established from a single natural population in Brisbane, Australia [41]. When the experiment began, the founding population had been maintained at The University of Queensland for approximately 200 generations at a large population size, on a 12 h : 12 h light : dark cycle at 25°C on a standard 1.5 carbohydrate (sucrose) : protein (yeast) (S : Y) diet. From this founding population, 20 replicate populations were established and randomly allocated in equal numbers to five dietary treatments (0.5, 1.0, 1.5, 2.0 and 2.5 S : Y) for 30 generations before the start of the experiment (specific diet characteristics are given in electronic supplementary material, table S1). Each dietary treatment consisted of four replicates: A, B, C and D. Each replicate contained four mixed-sex population bottles. To control for density, each new generation was established from 60 randomly selected virgin offspring from the previous generation. All the other environmental conditions were kept constant for each generation.

### 2.2. Experimental design

Two generations of common garden were used to remove common environmental and parental effects from the S : Y diet treatments before lifespan and survival was measured. All replicate experimental populations were reared back on the ancestral diet for two generations before phenotyping. The solid blue arrow in figure 2*c* represents the experimental set-up for examining the plastic responses to developmental diet, i.e. the transfer of the ancestral diet replicate populations lines to every other developmental diet. The dotted blue arrow represent the experimental set-up for examining the evolved changes, i.e. the transfer of replicates from every evolutionary diet back to the ancestral diet.

After two generations of common garden, virgin flies were collected using CO_2_, sorted by sex and transferred to single-sex experimental vials for the lifespan assay. For each evolutionary diet × developmental diet combination, we used 10 replicate vials with 10 flies per each of the four replicate bottles (*N* = 400). Sample sizes are given in electronic supplementary material, tables S2 and S3. Flies were maintained at 25°C and 60% humidity, on a 12 : 12 h light : dark cycle. Survival was checked every 3–4 days and flies were tipped into fresh food vials without anaesthesia. On these occasions, dead flies were counted and removed from vials without replacement. This continued until all flies had died. Flies that escaped while changing food vials were excluded from subsequent lifespan and survival analysis.

### 2.3. Statistical analysis

PROC GLIMMIX (SAS software v. 9.4) was used to analyse treatment effects on adult lifespan in *D. serrata* according to the split-split-plot experimental design outlined in Littell *et al*. [42]. We used a mixed effects linear model to analyse the evolved and plastic effects of diet on lifespan. Models were fit a categorical ‘diet' effect that included the five diets (0.5, 1.0, 1.5, 2.0 and 2.5 S : Y). Given the sex differences in *D. serrata* lifespan, different models were fit to examine the effects of evolutionary and developmental diets, with sex as a fixed effect to control for and detect any sex-specific effects.

The model for plastic responses contained *developmental diet* as the fixed effect,

2.1 $$\begin{array}{rl} L= & \mu \;+\mathrm{sex}+\mathrm{developmental}\;\mathrm{diet}\;+\mathrm{sex}\times \mathrm{developmental}\;\;\mathrm{diet}+\;\mathrm{replicate}\\ & +\mathrm{developmental}\;\mathrm{diet}\times \mathrm{replicate}+\mathrm{vial}(\mathrm{sex}\;\times \mathrm{replicate}\;\times \mathrm{developmental}\;\mathrm{diet})+\;\varepsilon ,\;\; \end{array}$$

where lifespan *L* is measured in days, *μ* is mean lifespan, developmental diet is the fixed main effect of diet, replicate is a random effect describing variation among the four replicate (control, 1.5 S : Y diet) populations from the evolution experiment and *ε* is the residual error.

The model for evolved responses contained *evolutionary diet* as a fixed effect,

2.2 $$\begin{array}{rl} L= & \;\mu \;+\mathrm{sex}+\mathrm{evolutionary}\;\mathrm{diet}\;+\mathrm{sex}\times \mathrm{evolutionary}\;\mathrm{diet}+\;\mathrm{replicate}(\mathrm{evolutionary}\;\mathrm{diet})\\ & +\mathrm{sex}\times \mathrm{replicate}(\mathrm{evolutionary}\;\mathrm{diet})+\mathrm{vial}(\mathrm{sex}\times \mathrm{replicate}\times \mathrm{evolutionary}\;\;\mathrm{diet})+\;\varepsilon ,\;\; \end{array}$$

where lifespan *L* is measured in days, *μ* is mean lifespan, evolutionary diet is the fixed main effect of diet, replicate(evolutionary diet) is a random effect of replicate population nested within diet and *ε* is the residual error.

For both models, we used a Satterthwaite approximation to calculate the denominator degrees of freedom via the ‘ddfm = SAT' option in SAS software when testing significance of the diet effect. We present effect sizes as least square means and used Tukey's honestly significant difference (HSD) to test for differences between specific diets (*α* = 0.05).

As a follow-up to validate the patterns of mean lifespan observed, we also carried out univariate/multivariate Cox proportional hazards regression analysis and generated Kaplan–Meier survival curves. Likelihood tests of differences in Cox proportional hazard ratios were used to measure differences in hazard, and comparisons of different Kaplan–Meier survival curves were performed using the log-rank test. In all cases, a *p* < 0.05 was considered statistically significant. All follow-up survival analyses were performed using GraphPad Prism version 10.0.

## 3. Results

### 3.1. Plasticity of lifespan in response to diet

**Figure 3.**
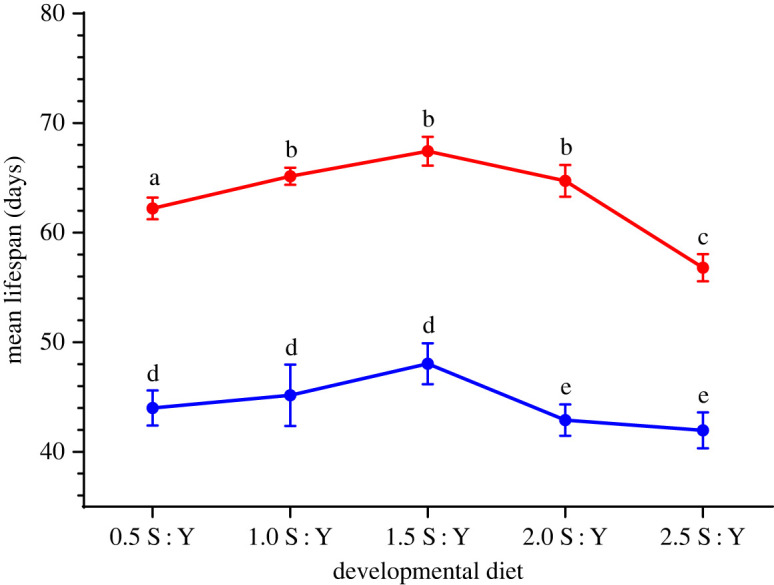
*Drosophila serrata* mean lifespan on developmental diets. Mean adult female (red bars) and male (blue bars) lifespan measured in days is shown for the 1.5 S : Y evolutionary diet transferred to every other developmental diet. Error bars represent the 1 s.e. of the mean. Letters indicate significance in planned comparisons between 1.5 S : Y control and novel diets within sexes.

We found that relative to the ancestral 1.5 S : Y diet, both high- and low-carbohydrate diets elicited plastic effects on lifespan. Lifespan responded to changes in nutritional environments for both sexes resulting in a bell-shaped relationship between lifespan and increasing carbohydrate content (figure 3). Lifespan responded to the novel diet treatments, with significant main effects of *sex* (*F*_1,371.6_ = 476.9*, p* = 1.32 × 10^−68^) and *developmental diet* (*F*_4,12.1_ = 6.54*, p* = 4.90 × 10^−03^) detected, but not *sex* × *developmental diet* (*F*_4,371.6_ = 1.82*, p* = 0.12) (figure 3). The plastic effects resemble a bell-shaped relationship between lifespan and increasing carbohydrate content with lifespan in both males and females maximized around the intermediate 1.5 S : Y developmental diet (figure 3). However, instead of increased lifespan on low S : Y diets as expected from decreasing sucrose content, there was a nonlinear relationship between developmental diets and lifespan (figure 3) that negatively affected adult survival (electronic supplementary material, figure S1). Compared with females, higher lifespan was observed over a broader range of low S : Y developmental diets for males, albeit with a tendency for lifespan to still peak on intermediate 1.5 S : Y developmental diets (figure 3).

Both high- and low-carbohydrate developmental diets significantly decreased longevity in female *D. serrata*. In females, *post hoc* comparisons relative to the 1.5 S : Y ancestral diet showed that flies raised on 0.5 and 2.5 S : Y developmental diets lived significantly shorter lives (0.5 S : Y versus 1.5 S : Y: *t* = −2.42, *p* = 0.02; 2.5 S : Y versus 1.5 S : Y: *t* = 4.90, *p* = 1.40 × 10^−05^; figure 3; electronic supplementary material, figure S1A and table S4). Neither mean lifespan nor survival was significantly different on the 1.0 and 2.0 S : Y developmental diets which were closer to the control 1.5 S : Y diet (1.0 S : Y versus 1.5 S : Y: *t* = −1.06, *p* = 0.30; 1.5 S : Y versus 2.0 S : Y: *t* = 1.26, *p* = 0.23; figure 3; electronic supplementary material, figure S1A and table S4). The significant plastic effects of developmental diets for lifespan could be driven by flies on both lower and higher S : Y diets living shorter and ageing faster than flies from populations closer to the ancestral 1.5 S : Y diet (figure 3; electronic supplementary material, figure S1A and table S8).

*Post hoc* comparisons for males showed that mean lifespan was not significantly different on any of the low S : Y developmental diets relative to the ancestral 1.5 S : Y control diet (0.5 S : Y versus 1.5 S : Y: *t* = −1.90, *p* = 0.07; 2.5 S : Y versus 1.5 S : Y: *t* = 0.95, *p* = 0.35; figure 3; electronic supplementary material, figure S1B; tables S2 and S5). However, compared with the 1.5 S : Y ancestral diet, male flies raised on 2.0 and 2.5 S : Y developmental diets lived significantly shorter lives with a higher risk of death (2.0 S : Y versus 1.5 S : Y: *t* = 2.41, *p* = 0.02; 2.5 S : Y versus 1.5 S : Y: *t* = 2.85, *p* = 0.01; figure 3; electronic supplementary material, figure S1B; table S2; S5 and S9).

### 3.2. Evolution of lifespan in response to diet

**Figure 4.**
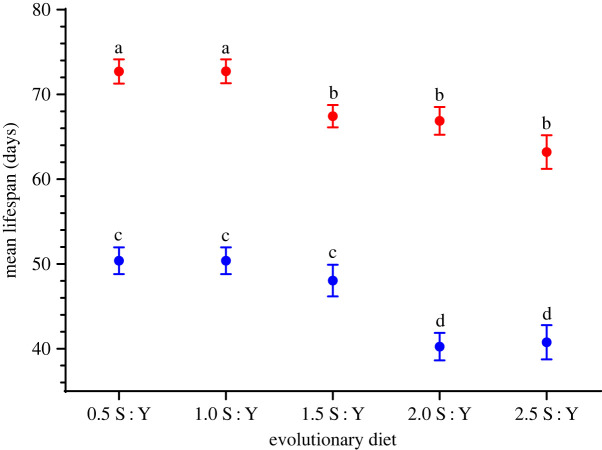
*Drosophila serrata* mean lifespan on evolutionary diets. Mean adult female (red bars) and male (blue bars) lifespan measured in days is shown for every evolutionary diet transferred to the 1.5 S : Y ancestral diet. Error bars represent the 1 s.e. of the mean. Letters indicate significance in planned comparisons between 1.5 S : Y control and novel diets.

Having detected lifespan plasticity in response to the developmental diets, we then tested whether those developmental plastic responses matched evolved responses to the same evolutionary diets. After approximately 30 generations of experimental evolution, we detected significant main effects of *evolutionary diet* (*F*_4,15.0_ = 10.2*, p* = 3.00 × 10^−04^) and *sex* (*F*_1,367.2_ = 544.8*, p* = 1.57 × 10^−74^), but not *sex* × *evolutionary diet* (*F*_4,367.2_ = 1.47*, p* = 0.21). With an overall negative relationship between lifespan and carbohydrate content, we found that both high- and low-carbohydrate diets elicited evolutionary effects on lifespan (figure 4). However, evolved responses to evolutionary diets did not resemble the pattern of plastic responses for lifespan on S : Y developmental diets.

*Post hoc* comparisons between females showed that flies evolved on lower S : Y diets of 0.5 and 1.0 S : Y lived on average 5.3, 5.8 and 9.5 days longer than flies evolved on 1.5, 2.0 and 2.5 S : Y diets, respectively (electronic supplementary material, table S3). Mean lifespan and survival time was significantly different on 0.5 and 1.0 S : Y diets (0.5 S : Y versus 1.5 S : Y: *t* = 2.05, *p* = 0.047; 1.0 S : Y versus 1.5 S : Y: *t* = 2.06, *p* = 0.046; figure 4; electronic supplementary material, figure S2A; tables S6 and S10). Mean lifespan was not significantly different on the 2.0 and 2.5 S : Y diet compared with the ancestral control diet of 1.5 S : Y (1.5 S : Y versus 2.0 S : Y: *t* = 0.25, *p* = 0.80; 1.5 S : Y versus 2.5 S : Y: *t* = 1.77, *p* = 0.09; figure 4; electronic supplementary material, figure S2A; tables S6 and S10). Female lifespan appeared more resilient to changes brought on by high S : Y evolution diets and evolution under high S : Y diets did not result in strong effects on female lifespan and survival patterns.

In contrast to females, *post hoc* comparisons showed that lifespan increases observed in male flies evolved on low sucrose diets were not statistically significant (0.5 S : Y versus 1.5 S : Y: *t* = 0.89, *p* = 0.38; 1.0 S : Y versus 1.5 S : Y: *t* = 0.38, *p* = 0.38; figure 4; electronic supplementary material, figure S2B; tables S7 and S11). Males on the ancestral 1.5 S : Y diet lived on average 8 days longer than males on higher S : Y diets (1.5 S : Y versus 2.0 S : Y: *t* = 3.19, *p* = 2.90 × 10^−03^; 1.5 S : Y versus 2.5 S : Y: *t* = 3.01, *p* = 4.60 × 10^−03^; figure 4; electronic supplementary material, figure S2B; table S3 and S5). Compared with females, males had less resilience to a high sucrose diet leading to a reduction in lifespan. The significant effects of novel diet for lifespan could be driven by flies on lower S : Y diets living longer and ageing slower than flies from higher S : Y diet populations (figure 4; electronic supplementary material, figure S2B, tables S3, S7 and S11).

## 4. Discussion

### 4.1. Dietary-based developmental plasticity affects lifespan

Our plasticity assay revealed a dietary optimum for male and female lifespan where developmental diet concentrations had a bell-shaped relationship with lifespan representative of the typical lifespan reaction norm to diet (figure 1). We did not detect any lifespan extension under low S : Y developmental diets in females or males. Instead, compared with the ancestral diet, we found decreased lifespan on low S : Y developmental diets. In contrast to our findings, Zajitschek *et al*. [20] found no difference in male fruit fly survivorship between evolution diet populations after approximately 25 generations. However, DR responses under protein manipulation are highly variable even among different genotypes of *D. melanogaster* with some genotypes showing no or only little lifespan extension on a DR diet, with others showing decreased lifespan on a DR diet [43]. Furthermore, we manipulated sucrose content while keeping yeast content the same, whereas most studies including Zajitschek *et al*. [20] and Zajitschek *et al*. [22] have manipulated yeast content. Studies have previously shown that varying the protein : carbohydrate balance, rather than the caloric content is the main driver of ageing processes and lifespan variation [39,44–46]. Decreasing either sucrose or dietary yeast extends lifespan and reduces mortality, but by an amount that is unrelated to the caloric content of the diet. Furthermore, diets higher in protein content have been shown to induce elevations in metabolic rate and reduce whole body carbohydrate content in adult *Drosophila* [47]. It should be acknowledged that we cannot distinguish the effects of the altered carbohydrate levels from that of an altered protein: carbohydrate ratio including vitamins, and other micronutrients in the yeast. It is possible that the plastic and evolved lifespan responses are driven by these changes, including the upregulation of gluconeogenesis and certain other metabolic pathways as well [48].

Under life-history theory predictions of decreased fitness and lifespan, the plastic response to low S : Y diets in our study most likely corresponds to a ‘stress' that is expressed when populations have not yet adapted to this new dietary environment [49]. For example, gene expression plasticity evolved in *D. melanogaster* adapted to a medium enriched in salt and cadmium [49], but the direction of plasticity observed was in the opposite direction compared with the ancestral populations. Salt and cadmium represent novel and extreme environments seldom encountered by fruit flies outside laboratory settings. Such extreme environments suggest a likelihood of maladaptive plasticity and therefore the possibility of robust selection [50,51], including on the plasticity of a trait [52]. A reason for not detecting lifespan extension plasticity on high sucrose diets in males might be that these diets represent stressful environments where the optimal lifespan phenotype is constrained, leaving females' lifespan more receptive to the effects of diets that are optimal for reproduction and lifespan [12,53,54]. Indeed, sites of fat deposition in females are different to those of males and have been proposed to reflect adaptations promoting female reproductive success [55]. An alternative hypothesis would be that the plastic responses are adaptions to a nutritional environment that is fluctuating or experienced transiently. Zajitschek *et al*. [22] employed experimental evolution to examine the ‘waiting-for-the-good-times' hypothesis. According to this hypothesis, selection during prolonged DR would encourage heightened reproductive investment at the expense of survival, as the anticipated ‘good times' never materialize. Contrary to expectations, their findings challenge the idea that reallocating resources between fecundity and somatic maintenance is the basis for extending lifespan under DR. However, we cannot exclude the possibility that the diet-induced changes might provide other adaptive benefits that trade off with lifespan and could not be measured easily under laboratory settings, e.g. greater reproductive investment associated with ranging for food that might allow for offspring to survive in poor environments and find more favourable conditions [56]. Finally, although both sexes can demonstrate nutritionally plastic responses, in many species it is the larger sex (females in flies) that is more nutritionally plastic [57], a pattern consistent with the present study.

### 4.2. Evolutionary adaptation to nutrient availability

Our experimental evolution experiments revealed that lifespan evolved in response to changes in dietary carbohydrate concentration and that the direction of the evolved response observed on low S : Y developmental diets was in the opposite direction to the plastic response. We found a strong positive effect of increased lifespan for evolution under low S : Y in females, whereas a prior experimental evolution study [22] found that females evolved under DR had lower lifespans compared with females that evolved on the standard diet. Another difference between this experimental evolution study and our study was that males evolved lower lifespans on high S : Y diets as opposed to no differences in male survivorship on any of the evolutionary diets in the previous study [20]. Flies evolved on higher carbohydrates, especially males, had shorter lifespans. According to previous studies, this suggests a preference or programme in adults where even after the carbohydrate challenge has been removed, lifelong exposure to diets high in carbohydrates may affect many metabolic pathways such as those involved in glucose homeostasis, or cholesterol metabolism in offspring [58,59].

Dietary interventions that extend lifespan can also be considered as a form of hormetic nutritional stress and may explain the potential to evolve longer lifespans [60,61]. In the context of ageing, DR is recognized as a nutritional hormetin due to its ability to trigger a stress response, leading to adaptive hormetic effects that ultimately result in physiological hormesis. This process is achieved by regulating stress chaperones, such as heat shock proteins (HSP70), antioxidants and redox enzyme activities. Overall, these contribute significantly to promoting cell and overall life longevity [62,63]. Although constant dietary excess such as diets high in sucrose have been known to accelerate ageing and reduce lifespan [18,40,64], it is still possible for such a diet to also select for longer lifespans given a particular combination of conditions [65,66]. Nutrient availability in response to distinct nutritional and metabolic demands of life-history strategies can cause organisms to experience alternative developmental programmes [67]. This enables organisms to endure severe or persistent environmental stress by altering physiological responses until environmental conditions improve [68,69] and may facilitate long-term adaptation [52]. For example, exposure to heat stress in *C. elegans* results in individuals with slower ageing [70]. Such adaptive responses may also have evolved to allow the females to survive periods of starvation during reproduction and would explain why female lifespan was more robust to the effects of a high carbohydrate evolutionary diet.

Although lifespan extension under DR is frequently attributed to energy reallocation for somatic maintenance instead of reproduction [71], the trade-offs sometimes observed between reproduction and lifespan are now thought to relate to hormonal signalling [72] or programmatic mechanisms [73] rather than a shift in reallocation of resources to somatic maintenance from reproduction. Experimental evolution studies have tested this resource reallocation prediction in male and female experimental evolution lines of *D. melanogaster* [20,22] and shown mixed and sex-specific results. Females evolved shorter lifespan but increased fecundity on DR [20], while males displayed increased fitness at no cost to lifespan on DR [22]. These findings show that lifespan can evolve in response to diet, but the results contradict the typical hypothesis that resource reallocation is driving lifespan extension as predicted under the disposable soma theory of ageing. An unexplored but important factor may be potential confounding due to selection by carbohydrate diets acting on larval traits such as survivorship, growth and body size that influence lifespan instead rather than acting directly on lifespan. In *Drosophila*, studies have used experimental evolution to study the impact of diet composition and shown that rearing under alternative diet regimes for multiple generations results in genetically based changes in life-history traits [20,22,74] but not lifespan. However, it is still possible that the evolved changes in lifespan found after 30 generations of selection could also be the indirect outcome of evolved changes in previously unmeasured larval traits that correlate with lifespan in *Drosophila*. Although an increasing number of experimental evolution studies now show that trade-offs between longevity and life-history traits are non-ubiquitous and can be uncoupled [20,22,74], diet-driven changes in lifespan associated with life-history traits, such as reproduction and body size may further advance our understanding of the evolution of dietary interventions in ageing and lifespan.

While examinations of fitness and lifespan trade-offs were beyond the scope of the present study, lifespan extensions have been shown to occur even in sterile and non-reproducing animals in the absence of trade-offs between reproduction and somatic maintenance [75]. While genotypic (female/male) and environmental (sucrose rich/sucrose poor) specific constraints might also restrict the evolution of phenotypes that are similar in both sexes [22] based on our experimental data, it is reasonable to assume that the effects observed in male and female *D. serrata* under experimental evolution represent similar diet-dependent patterns that have evolved but are different from the plastic lifespan patterns observed in the same populations.

## 5. Conclusion

We previously showed that lifespan variation in *D. serrata* arises due to condition-dependent environmental modulations of underlying genetic variation [76]. Dietary discordance between organisms' evolutionary and developmental history is increasingly recognized to play a critical role in shaping lifespan and survival [13,77,78]. While this study demonstrates that diets with varying levels of carbohydrates can have evolved and plastic effects on lifespan for *D. serrata* on the ancestral dietary environment, there was a pronounced misalignment between the plastic and evolved responses. The match/mismatch implies that plastic responses to high carbohydrates are adaptive but plastic responses to low carbohydrates are not. Here, we were only able to test the dietary treatments in a single environment to isolate the evolved response, but whether this holds true across a broader range of evolutionary and developmental diet combinations merits further investigation. This would allow us to test the evolved responses across all the different diets to see if/how plasticity evolves as a correlated response to selection imposed by the different dietary environments. Such match/mismatch approaches could improve the understanding of evolved and plastic responses in natural populations and subsequent consequences to health and lifespan. This approach also helps translate our understanding regarding the impact of contemporary diets into interventions aimed at optimizing or ameliorating population health and lifespan [79,80]. Ultimately, understanding the basis of this variation for lifespan and the different responses to dietary treatments across a broader range of evolutionary and developmental diets may help address the causes and consequences of rapidly changing dietary histories of human populations.

## Data Availability

Lifespan data: Dryad Digital Repository: https://doi.org/10.5061/dryad.ffbg79czt [81]. Supplementary material is available online [82].

## References

[1] Ocampo M, Pincheira-Donoso D, Sayol F, Rios RS. 2022 Evolutionary transitions in diet influence the exceptional diversification of a lizard adaptive radiation. BMC Ecol. Evol. **22**, 74. (10.1186/s12862-022-02028-3) 35672668 PMC9175459

[2] Zajitschek F, Jin T, Colchero F, Maklakov AA. 2014 Aging differently: diet- and sex-dependent late-life mortality patterns in *Drosophila melanogaster*. J. Gerontol. A Biol. Sci. Med. Sci. **69**, 666-674. (10.1093/gerona/glt158) 24170671

[3] Lee KP. 2015 Dietary protein:carbohydrate balance is a critical modulator of lifespan and reproduction in *Drosophila melanogaster*: a test using a chemically defined diet. J. Insect. Physiol. **75**, 12-19. (10.1016/j.jinsphys.2015.02.007) 25728576

[4] Archer CR, Basellini U, Hunt J, Simpson SJ, Lee KP, Baudisch A. 2018 Diet has independent effects on the pace and shape of aging in *Drosophila melanogaster*. Biogerontology **19**, 1-12. (10.1007/s10522-017-9729-1) 28914388 PMC5765211

[5] Jang T, Lee KP. 2018 Comparing the impacts of macronutrients on life-history traits in larval and adult *Drosophila melanogaster*: the use of nutritional geometry and chemically defined diets. J. Exp. Biol. **221**, jeb181115. (10.1242/jeb.181115) 30171098

[6] Min KW, Jang T, Lee KP. 2021 Thermal and nutritional environments during development exert different effects on adult reproductive success in *Drosophila melanogaster*. Ecol. Evol. **11**, 443-457. (10.1002/ece3.7064) 33437441 PMC7790642

[7] Barson NJ et al. 2015 Sex-dependent dominance at a single locus maintains variation in age at maturity in salmon. Nature **528**, 405-408. (10.1038/nature16062) 26536110

[8] Jensen K, McClure C, Priest NK, Hunt J. 2015 Sex-specific effects of protein and carbohydrate intake on reproduction but not lifespan in *Drosophila melanogaster*. Aging Cell **14**, 605-615. (10.1111/acel.12333) 25808180 PMC4531074

[9] Lihoreau M, Poissonnier LA, Isabel G, Dussutour A. 2016 *Drosophila* females trade off good nutrition with high-quality oviposition sites when choosing foods. J. Exp. Biol. **219**, 2514-2524. 27284071 10.1242/jeb.142257

[10] Camus MF, Fowler K, Piper MWD, Reuter M. 2017 Sex and genotype effects on nutrient-dependent fitness landscapes in *Drosophila melanogaster*. Proc. Biol. Sci. **284**, 20172237. (10.1098/rspb.2017.2237) 29263276 PMC5745421

[11] Brengdahl M, Kimber CM, Maguire-Baxter J, Malacrino A, Friberg U. 2018 Genetic quality affects the rate of male and female reproductive aging differently in *Drosophila melanogaster*. Am. Nat. **192**, 761-772. (10.1086/700117) 30444654

[12] Camus MF, Piper MD, Reuter M. 2019 Sex-specific transcriptomic responses to changes in the nutritional environment. Elife **8**, e47262. (10.7554/eLife.47262) 31436529 PMC6773443

[13] Duxbury EML, Chapman T. 2019 Sex-specific responses of life span and fitness to variation in developmental versus adult diets in *Drosophila melanogaster*. J. Gerontol. A Biol. Sci. Med. Sci. **75**, 1431-1438. (10.1093/gerona/glz175) PMC7357588 31362304

[14] Hosking CJ, Raubenheimer D, Charleston MA, Simpson SJ, Senior AM. 2019 Macronutrient intakes and the lifespan-fecundity trade-off: a geometric framework agent-based model. J. R. Soc. Interface **16**, 20180733. (10.1098/rsif.2018.0733) 30958189 PMC6408357

[15] Klepsatel P, Knoblochova D, Girish TN, Dircksen H, Galikova M. 2020 The influence of developmental diet on reproduction and metabolism in *Drosophila*. BMC Evol. Biol. **20**, 93. (10.1186/s12862-020-01663-y) 32727355 PMC7392729

[16] Magwere T, Chapman T, Partridge L. 2004 Sex differences in the effect of dietary restriction on life span and mortality rates in female and male *Drosophila melanogaster*. J. Gerontol. A Biol. Sci. Med. Sci. **59**, 3-9. (10.1093/gerona/59.1.B3) 14718480

[17] Kim K, Jang T, Min K-J, Lee KP. 2020 Effects of dietary protein:carbohydrate balance on life-history traits in six laboratory strains of *Drosophila melanogaster*. Entomol. Exp. Appl. **168**, 482-491. (10.1111/eea.12855)

[18] Dobson AJ, Ezcurra M, Flanagan CE, Summerfield AC, Piper MDW, Gems D, Alic N. 2017 Nutritional programming of lifespan by FOXO inhibition on sugar-rich diets. Cell Rep. **18**, 299-306. (10.1016/j.celrep.2016.12.029) 28076775 PMC5263231

[19] Öst A et al. 2014 Paternal diet defines offspring chromatin state and intergenerational obesity. Cell **159**, 1352-1364. (10.1016/j.cell.2014.11.005) 25480298

[20] Zajitschek F, Zajitschek SR, Canton C, Georgolopoulos G, Friberg U, Maklakov AA. 2016 Evolution under dietary restriction increases male reproductive performance without survival cost. Proc. Biol. Sci. **283**, 20152726. 26911958 10.1098/rspb.2015.2726 PMC4810831

[21] Emborski C, Mikheyev AS. 2019 Ancestral diet transgenerationally influences offspring in a parent-of-origin and sex-specific manner. Phil. Trans. R. Soc. B **374**, 20180181. (10.1098/rstb.2018.0181) 30966955 PMC6365861

[22] Zajitschek F, Georgolopoulos G, Vourlou A, Ericsson M, Zajitschek SRK, Friberg U, Maklakov AA. 2019 Evolution under dietary restriction decouples survival from fecundity in *Drosophila melanogaster* females. J. Gerontol. A Biol. Sci. Med. Sci. **74**, 1542-1548. (10.1093/gerona/gly070) 29718269

[23] Furse S, Watkins AJ, Hojat N, Smith J, Williams HEL, Chiarugi D, Koulman A. 2021 Lipid traffic analysis reveals the impact of high paternal carbohydrate intake on offsprings' lipid metabolism. Commun. Biol. **4**, 163. (10.1038/s42003-021-01686-1) 33547386 PMC7864968

[24] Ivimey-Cook ER, Sales K, Carlsson H, Immler S, Chapman T, Maklakov AA. 2021 Transgenerational fitness effects of lifespan extension by dietary restriction in *Caenorhabditis elegans*. Proc. Biol. Sci. **288**, 20210701. 33975472 10.1098/rspb.2021.0701 PMC8113902

[25] Pigliucci M, Murren CJ, Schlichting CD. 2006 Phenotypic plasticity and evolution by genetic assimilation. J. Exp. Biol. **209**, 2362-2367. (10.1242/jeb.02070) 16731812

[26] Ghalambor CK, McKay JK, Carroll SP, Reznick DN. 2007 Adaptive versus non-adaptive phenotypic plasticity and the potential for contemporary adaptation in new environments. Funct. Ecol. **21**, 394-407. (10.1111/j.1365-2435.2007.01283.x)

[27] Chevin L-M, Lande R, Mace GM. 2010 Adaptation, plasticity, and extinction in a changing environment: towards a predictive theory. PLoS Biol. **8**, e1000357. (10.1371/journal.pbio.1000357) 20463950 PMC2864732

[28] Vinton AC, Gascoigne SJL, Sepil I, Salguero-Gomez R. 2022 Plasticity's role in adaptive evolution depends on environmental change components. Trends Ecol. Evol. **37**, 1067-1078. (10.1016/j.tree.2022.08.008) 36153155

[29] Grether GF. 2005 Environmental change, phenotypic plasticity, and genetic compensation. Am. Nat. **166**, E115-E123. (10.1086/432023) 16224697

[30] Schaum CE, Collins S. 2014 Plasticity predicts evolution in a marine alga. Proc. Biol. Sci. **281**, 20141486. (10.1098/rspb.2014.1486) 25209938 PMC4173685

[31] Lucas A. 1998 Programming by early nutrition: an experimental approach. J. Nutr. **128**, 401S-406S. (10.1093/jn/128.2.401S) 9478036

[32] Baugh LR, Day T. 2020 Nongenetic inheritance and multigenerational plasticity in the nematode *C. elegans*. Elife **9**, e58498. (10.7554/eLife.58498) 32840479 PMC7447421

[33] Mair W, Goymer P, Pletcher SD, Partridge L. 2003 Demography of dietary restriction and death in *Drosophila*. Science **301**, 1731-1733. (10.1126/science.1086016) 14500985

[34] Simons MJ, Koch W, Verhulst S. 2013 Dietary restriction of rodents decreases aging rate without affecting initial mortality rate – a meta-analysis. Aging Cell **12**, 410-414. (10.1111/acel.12061) 23438200

[35] McCracken AW, Buckle E, Simons MJP. 2020 The relationship between longevity and diet is genotype dependent and sensitive to desiccation in *Drosophila melanogaster*. J. Exp. Biol. **223**, jeb230185. (10.1242/jeb.230185) 33109715 PMC7725603

[36] Cordain L, Eaton SB, Sebastian A, Mann N, Lindeberg S, Watkins BA, O'Keefe JH, Brand-Miller J. 2005 Origins and evolution of the Western diet: health implications for the 21st century. Am. J. Clin. Nutr. **81**, 341-354. (10.1093/ajcn.81.2.341) 15699220

[37] Luca F, Perry GH, Di Rienzo A. 2010 Evolutionary adaptations to dietary changes. Annu. Rev. Nutr. **30**, 291-314. (10.1146/annurev-nutr-080508-141048) 20420525 PMC4163920

[38] Lea AJ, Martins D, Kamau J, Gurven M, Ayroles JF. 2020 Urbanization and market integration have strong, nonlinear effects on cardiometabolic health in the Turkana. Sci. Adv. **6**, eabb1430. (10.1126/sciadv.abb1430) 33087362 PMC7577730

[39] Skorupa DA, Dervisefendic A, Zwiener J, Pletcher SD. 2008 Dietary composition specifies consumption, obesity, and lifespan in *Drosophila melanogaster*. Aging Cell **7**, 478-490. (10.1111/j.1474-9726.2008.00400.x) 18485125 PMC2574586

[40] Chandegra B, Tang JLY, Chi H, Alic N. 2017 Sexually dimorphic effects of dietary sugar on lifespan, feeding and starvation resistance in *Drosophila*. Aging **9**, 2521-2528. (10.18632/aging.101335) 29207375 PMC5764390

[41] Reddiex AJ, Allen SL, Chenoweth SF. 2018 A genomic reference panel for *Drosophila serrata*. G3 (Bethesda) **8**, 1335-1346. (10.1534/g3.117.300487) 29487184 PMC5873922

[42] Littell RC, Milliken GA, Stroup WW, Wolfinger RD, Oliver S. 2006 SAS for mixed models. Cary, NC: SAS publishing.

[43] Stanley PD, Ng'oma E, O'Day S, King EG. 2017 Genetic dissection of nutrition-induced plasticity in insulin/insulin-like growth factor signaling and median life span in a *Drosophila* multiparent population. Genetics **206**, 587-602. (10.1534/genetics.116.197780) 28592498 PMC5499174

[44] Mair W, Piper MD, Partridge L. 2005 Calories do not explain extension of life span by dietary restriction in *Drosophila*. PLoS Biol. **3**, e223. (10.1371/journal.pbio.0030223) 16000018 PMC1140680

[45] Grandison RC, Piper MD, Partridge L. 2009 Amino-acid imbalance explains extension of lifespan by dietary restriction in *Drosophila*. Nature **462**, 1061-1064. (10.1038/nature08619) 19956092 PMC2798000

[46] Tatar M, Post S, Yu K. 2014 Nutrient control of *Drosophila* longevity. Trends Endocrinol. Metab. **25**, 509-517. (10.1016/j.tem.2014.02.006) 24685228 PMC4177520

[47] Bradley TJ, Simmons FH. 1997 An analysis of resource allocation in response to dietary yeast in *Drosophila melanogaster*. J. Insect. Physiol. **43**, 779-788. (10.1016/S0022-1910(97)00037-1) 12770456

[48] Winwood-Smith HS, Franklin CE, White CR. 2017 Low-carbohydrate diet induces metabolic depression: a possible mechanism to conserve glycogen. Am. J. Physiol. Regul. Integr. Comp. Physiol. **313**, R347-R356. (10.1152/ajpregu.00067.2017) 28701319

[49] Huang Y, Agrawal AF. 2016 Experimental evolution of gene expression and plasticity in alternative selective regimes. PLoS Genet. **12**, e1006336. (10.1371/journal.pgen.1006336) 27661078 PMC5035091

[50] Hoffmann AA, Hercus MJ. 2000 Environmental stress as an evolutionary force. Bioscience **50**, 217-226. (10.1641/0006-3568(2000)050[0217:ESAAEF]2.3.CO;2)

[51] Agrawal AF, Whitlock MC. 2010 Environmental duress and epistasis: how does stress affect the strength of selection on new mutations? Trends Ecol. Evol. **25**, 450-458. (10.1016/j.tree.2010.05.003) 20538366

[52] Lande R. 2009 Adaptation to an extraordinary environment by evolution of phenotypic plasticity and genetic assimilation. J. Evol. Biol. **22**, 1435-1446. (10.1111/j.1420-9101.2009.01754.x) 19467134

[53] Rapkin J, Jensen K, Archer CR, House CM, Sakaluk SK, Castillo ED, Hunt J. 2018 The geometry of nutrient space-based life-history trade-offs: sex-specific effects of macronutrient intake on the trade-off between encapsulation ability and reproductive effort in decorated crickets. Am. Nat. **191**, 452-474. (10.1086/696147) 29570407

[54] Kim KE, Jang T, Lee KP. 2020 Combined effects of temperature and macronutrient balance on life-history traits in *Drosophila melanogaster*: implications for life-history trade-offs and fundamental niche. Oecologia **193**, 299-309. (10.1007/s00442-020-04666-0) 32418116

[55] Power ML, Schulkin J. 2008 Sex differences in fat storage, fat metabolism, and the health risks from obesity: possible evolutionary origins. Br. J. Nutr. **99**, 931-940. (10.1017/S0007114507853347) 17977473

[56] Pontzer H, Kamilar JM. 2009 Great ranging associated with greater reproductive investment in mammals. Proc. Natl Acad. Sci. USA **106**, 192-196. (10.1073/pnas.0806105106) 19109432 PMC2629235

[57] Rohner PT, Teder T, Esperk T, Lüpold S, Blanckenhorn WU. 2018 The evolution of male-biased sexual size dimorphism is associated with increased body size plasticity in males. Funct. Ecol. **32**, 581-591. (10.1111/1365-2435.13004)

[58] Ormerod KG, LePine OK, Abbineni PS, Bridgeman JM, Coorssen JR, Mercier AJ, Tattersall GJ. 2017 *Drosophila* development, physiology, behavior, and lifespan are influenced by altered dietary composition. Fly (Austin) **11**, 153-170. (10.1080/19336934.2017.1304331) 28277941 PMC5552271

[59] Wu Q et al. 2020 Sexual dimorphism in the nutritional requirement for adult lifespan in *Drosophila melanogaster*. Aging Cell **19**, e13120. (10.1111/acel.13120) 32069521 PMC7059147

[60] Sinclair DA. 2005 Toward a unified theory of caloric restriction and longevity regulation. Mech. Ageing Dev. **126**, 987-1002. (10.1016/j.mad.2005.03.019) 15893363

[61] Rattan SI, Fernandes RA, Demirovic D, Dymek B, Lima CF. 2009 Heat stress and hormetin-induced hormesis in human cells: effects on aging, wound healing, angiogenesis, and differentiation. Dose Response **7**, 90-103. (10.2203/dose-response.08-014.Rattan) 19343114 PMC2664638

[62] Calabrese EJ. 2014 Hormesis: a fundamental concept in biology. Microbial Cell **1**, 145. (10.15698/mic2014.05.145) 28357236 PMC5354598

[63] Sharma A, Kaur T, Singh H, Kaur G. 2019 Chapter 8: Intermittent fasting–dietary restriction as a biological hormetin for health benefits. In The science of hormesis in health and longevity (eds SIS Rattan, M Kyriazis), pp. 99-104. New York, NY: Academic Press.

[64] Giugliano D, Maiorino MI, Bellastella G, Esposito K. 2018 More sugar? No, thank you! The elusive nature of low carbohydrate diets. Endocrine **61**, 383-387. (10.1007/s12020-018-1580-x) 29556949

[65] Bruce KD et al. 2013 High carbohydrate-low protein consumption maximizes *Drosophila* lifespan. Exp. Gerontol. **48**, 1129-1135. (10.1016/j.exger.2013.02.003) 23403040 PMC3687007

[66] Shokhirev MN, Johnson AA. 2014 Effects of extrinsic mortality on the evolution of aging: a stochastic modeling approach. PLoS ONE **9**, e86602. (10.1371/journal.pone.0086602) 24466165 PMC3897743

[67] Lafuente E, Beldade P. 2019 Genomics of developmental plasticity in animals. Front. Genet. **10**, 720. (10.3389/fgene.2019.00720) 31481970 PMC6709652

[68] Braendle C, Félix M-A. 2008 Plasticity and errors of a robust developmental system in different environments. Dev. Cell **15**, 714-724. (10.1016/j.devcel.2008.09.011) 19000836

[69] Sommer RJ. 2020 Phenotypic plasticity: from theory and genetics to current and future challenges. Genetics **215**, 1-13. (10.1534/genetics.120.303163) 32371438 PMC7198268

[70] Chen HY, Maklakov AA. 2012 Longer life span evolves under high rates of condition-dependent mortality. Curr. Biol. **22**, 2140-2143. (10.1016/j.cub.2012.09.021) 23084993

[71] Kirkwood TB. 1977 Evolution of ageing. Nature **270**, 301-304. (10.1038/270301a0) 593350

[72] Adler MI, Cassidy EJ, Fricke C, Bonduriansky R. 2013 The lifespan-reproduction trade-off under dietary restriction is sex-specific and context-dependent. Exp. Gerontol. **48**, 539-548. (10.1016/j.exger.2013.03.007) 23542072

[73] Gems D. 2022 The hyperfunction theory: an emerging paradigm for the biology of aging. Ageing Res. Rev. **74**, 101557. (10.1016/j.arr.2021.101557) 34990845 PMC7612201

[74] Kolss M, Vijendravarma RK, Schwaller G, Kawecki TJ. 2009 Life-history consequences of adaptation to larval nutritional stress in *Drosophila*. Evolution **63**, 2389-2401. (10.1111/j.1558-5646.2009.00718.x) 19473389

[75] Ghazi A, Henis-Korenblit S, Kenyon C. 2009 A transcription elongation factor that links signals from the reproductive system to lifespan extension in *Caenorhabditis elegans*. PLoS Genet. **5**, e1000639. (10.1371/journal.pgen.1000639) 19749979 PMC2729384

[76] Narayan VP, Wilson AJ, Chenoweth SF. 2022 Genetic and social contributions to sex differences in lifespan in *Drosophila serrata*. J. Evol. Biol. **35**, 657-663. (10.1111/jeb.13992) 35290690 PMC9314142

[77] Raubenheimer D, Simpson SJ, Tait AH. 2012 Match and mismatch: conservation physiology, nutritional ecology and the timescales of biological adaptation. Phil. Trans. R. Soc. B **367**, 1628-1646. (10.1098/rstb.2012.0007) 22566672 PMC3350655

[78] Feige-Diller J, Palme R, Kaiser S, Sachser N, Richter SH. 2021 The impact of varying food availability on health and welfare in mice: testing the Match-Mismatch hypothesis. Physiol. Behav. **228**, 113193. (10.1016/j.physbeh.2020.113193) 33011232

[79] Monaghan P. 2008 Early growth conditions, phenotypic development and environmental change. Phil. Trans. R. Soc. B **363**, 1635-1645. (10.1098/rstb.2007.0011) 18048301 PMC2606729

[80] Tilman D, Clark M. 2014 Global diets link environmental sustainability and human health. Nature **515**, 518-522. (10.1038/nature13959) 25383533

[81] Narayan VP, Wasana N, Wilson AJ, Chenoweth SF. 2024 Misalignment of plastic and evolutionary responses of lifespan to novel carbohydrate diets. Dryad Digital Repository. (10.5061/dryad.ffbg79czt) PMC10791524 38234441

[82] Narayan VP, Wasana N, Wilson AJ, Chenoweth SF. 2024 Misalignment of plastic and evolutionary responses of lifespan to novel carbohydrate diets. Figshare. (10.6084/m9.figshare.c.7027743) PMC10791524 38234441

